# The Role of Metabolic Lipases in the Pathogenesis and Management of Liver Disease

**DOI:** 10.1002/hep.31250

**Published:** 2020-09-28

**Authors:** Matteo Tardelli, Francesca Virginia Bruschi, Michael Trauner

**Affiliations:** ^1^ Hans Popper Laboratory of Molecular Hepatology Division of Gastroenterology and Hepatology Department of Medicine III Medical University of Vienna Vienna Austria; ^2^ Division of Gastroenterology and Hepatology Joan and Sanford I. Weill Cornell Department of Medicine Weill Cornell Medical College New York NY

## Abstract

Intracellular lipolysis is an enzymatic pathway responsible for the catabolism of triglycerides (TGs) that is complemented by lipophagy as the autophagic breakdown of lipid droplets. The hydrolytic cleavage of TGs generates free fatty acids (FFAs), which can serve as energy substrates, precursors for lipid synthesis, and mediators in cell signaling. Despite the fundamental and physiological importance of FFAs, an oversupply can trigger lipotoxicity with impaired membrane function, endoplasmic reticulum stress, mitochondrial dysfunction, cell death, and inflammation. Conversely, impaired release of FFAs and other lipid mediators can also disrupt key cellular signaling functions that regulate metabolism and inflammatory processes. This review will focus on specific functions of intracellular lipases in lipid partitioning, covering basic and translational findings in the context of liver disease. In addition, the clinical relevance of genetic mutations in human disease and potential therapeutic opportunities will be discussed.

**Abbreviations**
ALD: alcoholic liver disease
ASO: antisense oligonucleotide
ATGL: adipose triglyceride lipase
BAT: brown adipose tissue
C: cytosine
CES: carboxylesterase
CGI‐58: comparative gene identification‐58
DG: diacylglycerol
ER: endoplasmic reticulum
FFA: free fatty acid
G: guanine
HCC: hepatocellular carcinoma
HCV: hepatitis C virus
HFD: high‐fat diet
HSC: hepatic stellate cell
HSL: hormone‐sensitive lipase
LD: lipid droplet
LXR: liver X receptor
MG: monoacylglycerol
MGL: MG lipase
NAFLD: nonalcoholic fatty liver disease
NASH: nonalcoholic steatohepatitis
NR: nuclear receptor
PNPLA: patatin‐like phospholipase domain–containing protein
PPAR: peroxisome proliferator–activated receptor
PUFA: polyunsaturated fatty acid
SNP: single‐nucleotide polymorphism
SREBP: regulatory element–binding protein
TG: triglyceride
VLDL: very low density lipoprotein

Triglycerides (TGs) represent the neutral energy storage of eukaryotic cells. Degradation of cytoplasmic TGs through hydrolytic cleavage by intracellular lipases in a process termed *neutral lipolysis* results in release of free fatty acids (FFAs), which are indispensable molecules for fuel cellular catabolism, as well as synthesis of phospholipids, and serve as signaling molecules in distinct intracellular pathways.^(^ ^1^ ^)^ Lipid absorption involves hydrolysis of dietary fat by pancreatic lipases and bile acids (BAs) in the intestinal lumen, followed by the uptake of hydrolyzed products such as FFAs by enterocytes. Subsequently, lipids are resynthesized in the endoplasmic reticulum (ER) and secreted with chylomicrons containing mainly TGs but also cholesterol and phospholipids.^(^ ^2^ ^)^ Lipoprotein‐associated TGs are thereby hydrolyzed by lipoprotein lipase that provides FFAs for tissue use. In addition, lipophagy also facilitates the catabolism of cytoplasmic lipid‐droplet (LD) components to lysosomes for subsequent degradation by lysosomal enzymes.^(^ ^3^ ^)^ Lysosomal acid lipase also plays a crucial role in lipoprotein lipid catabolism and hydrolyzes cholesteryl esters and TGs in the cell.^(^ ^4^ ^)^ In humans, loss‐of‐function mutations of the lipase A gene cause rare lysosomal disorders, such as Wolman disease.^(^ ^4^ ^)^

Notably, FFAs, cholesterol, and its derivates, such as BAs, act as agonistic ligands for several nuclear receptors (NRs), such as peroxisome proliferator–activated receptors (PPARs), liver X receptor (LXR), farnesoid X receptor (FXR), and other key transcription factors, such as sterol regulatory element–binding proteins (SREBPs).^(^ ^5^ ^)^ Three main enzymes have been implicated in the complete intracellular hydrolysis of TGs in cellular lipid stores. Adipose TG lipase (ATGL) performs the first and rate‐limiting step of hydrolyzing TGs.^(^ ^3^ ^)^ This process is enhanced by perilipin and comparative gene identification‐58 (CGI‐58, also named a/b hydrolase domain–containing protein‐5),^(^ ^3^ ^)^ which expose the TG core of LDs to lipases and therefore enhances their activity. Hormone‐sensitive lipase (HSL) is a multifunctional enzyme capable of hydrolyzing a variety of acyl esters, including TGs, diacylglycerols (DGs), and monoacylglycerols (MGs).^(^ ^3^ ^)^ Finally, MG lipase (MGL) efficiently cleaves MGs into glycerol and FFAs (Fig. 1). The patatin‐like phospholipase domain–containing protein (PNPLA) family comprises nine human and eight murine members,^(^ ^6^ ^)^ including ATGL (also known as PNPLA2). PNPLA3 has shown indirect effects on lipid metabolism by interacting with and sequestering CGI‐58, thus limiting the access to LDs by ATGL or other lipase members.^(^ ^7^ ^)^ The physiological activity of ATGL, HSL, and MGL is regulated by the interaction with different proteins and coactivators, which ensure the adjustment/regulation of lipolytic rates to metabolic needs.^(^ ^1^, ^3^ ^)^

**FIG. 1:**
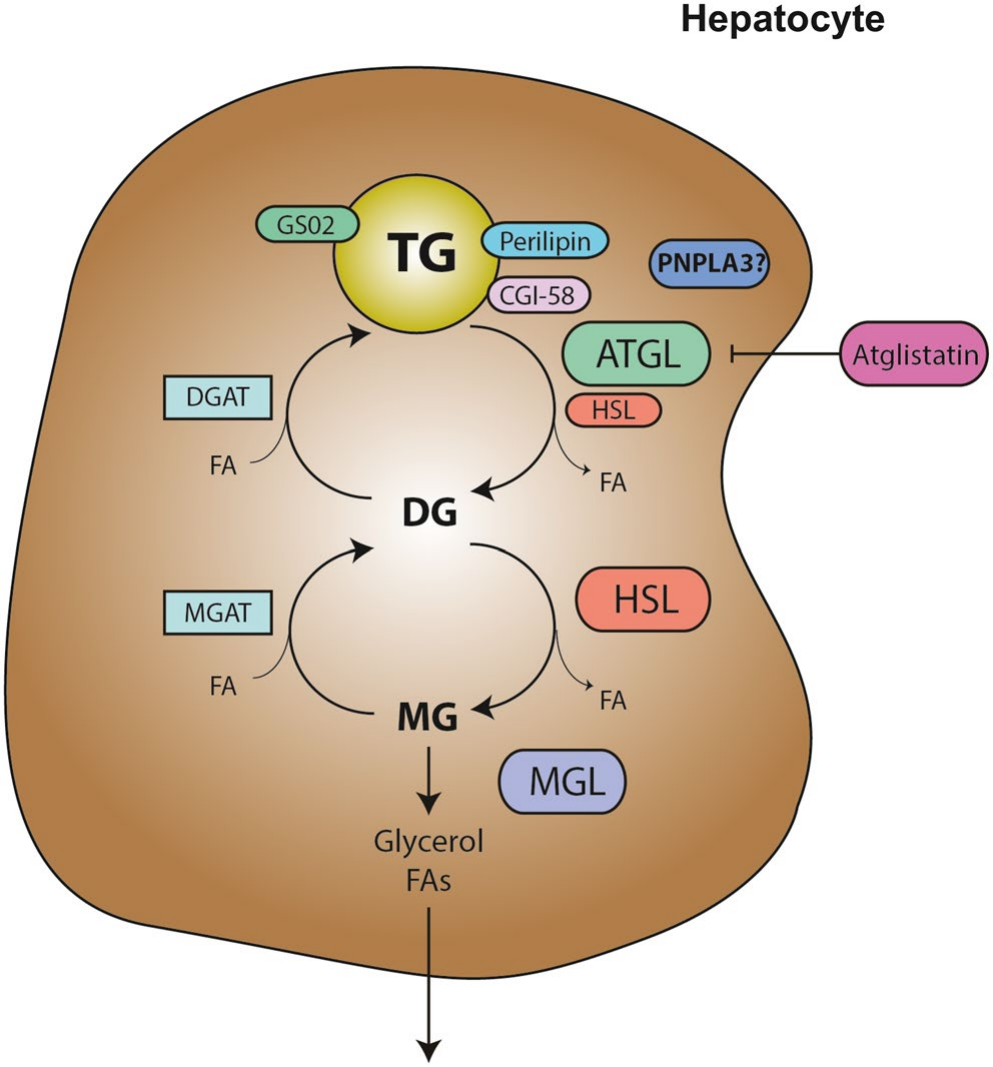
Catabolic pathways regulating TG levels in cells. ATGL, HSL, and MGL act on TGs in a series of subsequent enzymatic reactions, resulting in glycerol and FAs. CGI‐58 binds to perilipin, facilitating lipolysis as a known activator of ATGL, whereas *GS02* inhibits its action. Intermediates of this catabolic pathway may also be recycled for phospholipids and TG synthesis. In fact, significant amounts of hydrolysis products are normally re‐esterified by enzymes such as MGAT and DGAT. Abbreviations: ATGL, adipose triglyceride lipase; CGI‐58, comparative gene identification‐58; DGAT, FA, fatty acid; diacylglycerol acyltransferase; *GS02*, G_0_/G_1_ switch gene 2; HSL, hormone‐sensitive lipase; MGAT, monoacylglycerol acyltransferase; MGL, monoacylglycerol lipase; TG, triglyceride.

## ATGL/PNPLA2 is the Gatekeeper of Lipid Hydrolysis

Because mice with HSL deficiency did not show completely abolished lipolysis and had neither obesity nor cold sensitivity,^(^ ^8^ ^)^ it was indicated that HSL was not the only enzyme catalyzing TGs, which subsequently led to the discovery of ATGL.^(^ ^8^ ^)^ ATGL was shown to selectively perform the first step in TG hydrolysis, in which TGs are hydrolyzed to DGs and FFAs. For maximal lipolytic activity, ATGL requires CGI‐58 as a coactivator.^(^ ^9^ ^)^ Notably, CGI‐58 itself is also able to regulate hepatic neutral lipid storage (and inflammation), even in the genetic absence of ATGL.^(^ ^10^ ^)^ Several other proteins have also been shown to regulate ATGL enzymatic activity, such as adipocyte differentiation–related protein and G_0_/G_1_ switch gene 2 (G0S2).^(^ ^11^ ^)^ Strikingly, G0S2 was demonstrated to potently inhibit ATGL activity, also after CGI‐58 activation.^(^ ^12^ ^)^

ATGL is expressed in most tissues, with the highest mRNA levels and enzyme activity found in white adipose tissue (WAT) and brown adipose tissue (BAT).^(^ ^9^ ^)^ In mice lacking ATGL (*Atgl* ^−/−^), a great accumulation of TGs is observed in the heart, causing cardiac dysfunction and premature death.^(^ ^13^ ^)^ Intriguingly, ATGL deficiency was also shown to affect cold adaptation in BAT, insulin sensitivity, and glucose tolerance because of the reduced availability of FFAs as an energy substrate.^(^ ^13^ ^)^

In the liver, *Atgl* ^−/−^ mice show an enriched hepatic pool of oleic acid, which in turn prevented against pharmacologically induced (tunicamycin) ER stress.^(^ ^14^ ^)^ Ong et al.^(^ ^15^ ^)^ showed that ATGL deficiency leads to hepatic steatosis, concomitantly enhancing glucose tolerance and hepatic glucose use. Moreover, hepatic ATGL fuels FFAs necessary to stimulate PPARα activity, therefore positively regulating mitochondrial β‐oxidation.^(^ ^16^ ^)^ Consistent with these findings, hepatic mitochondrial oxidation was shown to be down‐regulated in high‐fat diet (HFD)‐fed mice receiving ATGL short hairpin RNA (shRNA) adenovirus.^(^ ^15^ ^)^ In addition, treatment with a PPARα agonist (fenofibrate) could not overcome decreased expression of PPARα target genes in mice treated with ATGL shRNA, suggesting that in liver, ATGL regulates PPARα through a ligand‐independent mechanism.^(^ ^15^ ^)^ Collectively, several studies have confirmed that the absence of hepatic ATGL results in liver steatosis without inflammation, down‐regulation of PPARα target genes, and reduced β‐oxidation, with improved hepatic glucose tolerance but normal insulin signaling.^(^ ^13^, ^15^ ^)^ *Atgl* ^−/−^ mice challenged with a methionine‐choline–deficient (MCD) diet or a lipopolysaccharide (LPS) to resemble features of nonalcoholic steatohepatitis (NASH) and hepatic inflammation, respectively, showed exacerbated hepatic steatosis and inflammation, which could be rescued by fibrate treatment in both models.^(^ ^17^ ^)^ This finding stands in contrast with those of the above‐mentioned study,^(^ ^14^ ^)^ a discrepancy that could be due to different doses of fenofibrate used and/or distinct fasting periods.^(^ ^15^ ^)^ Pharmacological inhibition of ATGL (with ATGL statin) was shown to ameliorate HFD‐induced insulin resistance, diminishing weight gain and steatosis in mice.^(^ ^18^ ^)^ Interestingly, *Cgi‐58* ^−/−^ mice die shortly after birth because of a skin defect. When CGI‐58 was knocked down in adult animals with antisense oligonucleotides (ASOs), liver TGs were raised 4‐fold,^(^ ^19^ ^)^ and mice were protected against diet‐induced obesity (DIO), despite the presence of hepatic steatosis.^(^ ^19^ ^)^ Collectively these findings suggest that pharmacological inhibition of ATGL and/or stimulation of PPARα signaling may have beneficial metabolic effects.

Several studies have investigated the clinical relevance of lipases, especially regarding rare genetic variants, such as single‐nucleotide polymorphisms (SNPs), which may influence pivotal lipid‐related traits. Importantly, *ATGL* genetic variants such as p.D244fs showed loss of function, whereas p.R79Q, p.R113H, and p.V402I presented markedly impaired catalytic activities.^(^ ^20^ ^)^ In another study, two heterozygous mutations, c.497A>G in exon 5 and c.1442C>T in exon 10, were reported to be associated with neutral lipid‐storage disease accompanied by myopathy weakness and fatigue.^(^ ^21^ ^)^ In addition, *CGI‐58* was found to be the causative gene for Chanarin‐Dorfman syndrome, a very rare neutral lipid‐storage disorder associated with ichthyosis, which affects multiple organs, including the liver, with steatosis or hepatomegaly.^(^ ^22^ ^)^

## PNPLA3 and its Potential Role in Lipid Metabolism

PNPLA3 (also known as adiponutrin) belongs to the same group of lipid‐metabolizing enzymes as ATGL (PNPLA2).^(^ ^6^ ^)^ Biochemical analysis reported a tight association of PNPLA3 with membranes and LDs.^(^ ^23^ ^)^ Despite considerable evidence on its clinical implications collected from its discovery in 2001,^(^ ^24^ ^)^ the enzymatic role of PNPLA3 still remains unclear. On one hand, it was shown that human recombinant PNPLA3 catalyzes the hydrolysis of TGs, DGs, and MGs (with higher preference for TGs)^(^ ^24^ ^)^; on the other hand, both human and murine PNPLA3 were described to function as acyl‐coenzyme A–dependent lysophosphatidic‐acid acyltransferase, thus favoring the synthesis of phosphatidic acid toward TGs.^(^ ^25^ ^)^ *In vitro* experiments on hepatocytes showed that PNPLA3 expression is promoted by glucose and insulin through SREBP‐1c, which subsequently controls expression of pivotal enzymes involved in *de novo* lipogenesis.^(^ ^26^ ^)^ SREBP‐1c binds on the promoter of *PNPLA3*, and overexpression of SREBP‐1c in hepatocytes therefore leads to increased *PNPLA3* gene expression.^(^ ^27^ ^)^ In addition, products of *de novo* lipogenesis (e.g., FFAs) enhance PNPLA3 protein half‐life, protecting it from proteasomal degradation.^(^ ^27^ ^)^ In contrast to *in vitro* findings, *in vivo* knockout strategies failed to elucidate its metabolic role, as mice lacking PNPLA3 displayed no phenotypic or metabolic alterations, despite several metabolic challenges (i.e., high‐sucrose diet, DIO, or MCD diet).^(^ ^28^ ^)^ Furthermore, no compensatory effect on other TG lipases or lipogenic enzymes has been reported in *Pnpla3* ^−/−^ mice, suggesting a nonexclusive role of this protein in TG metabolism *in vivo*.

Importantly, the *PNPLA3* I148M genetic variant creates a strong predisposition to developing nonalcoholic fatty liver disease (NAFLD) and alcoholic liver disease (ALD) toward steatohepatitis, fibrosis/cirrhosis, and liver cancer.^(^ ^29^ ^)^ This genetic variant consists of one single‐base polymorphism, where a cytosine (C) is substituted with a guanine (G), leading to a different amino acid sequence in the translated protein (methionine instead of isoleucine at residue 148). Several studies have reported a strong association between *PNPLA3* I148M and virtually every cause of liver disease.^(^ ^30^ ^)^

Moreover, the I148M polymorphism has been shown to promote cirrhosis and hepatocellular carcinoma (HCC) on NAFLD and ALD backgrounds.^(^ ^31^ ^)^ In regard to NAFLD‐related HCC, a multivariate analysis performed in a cross‐sectional study between European Caucasian patients reported that the CG and GG genotypes had a 2‐fold and 5‐fold increased risk for HCC, respectively, in comparison with the CC genotype.^(^ ^31^ ^)^ Recently, a prospective study on 471 patients with histologically diagnosed NAFLD reported an independent association between the *PNPLA3* C>G variant and HCC.^(^ ^32^ ^)^ Moreover, the I148M polymorphism has been shown to promote fibrosis and HCC in patients with chronic hepatitis C virus (HCV),^(^ ^33^ ^)^ and it is considered a risk factor for individuals co‐infected with HCV and human immunodeficiency virus, as these patients display a higher prevalence and occurrence of cirrhosis.^(^ ^34^ ^)^ This may have important implications for risk stratification for HCC screening in the precirrhosis stage. Significant correlations were found between severe steatosis and *PNPLA3* I148M in patients with concomitance of hepatitis B virus, obesity, and a history of alcohol intake,^(^ ^35^ ^)^ whereas in patients affected by primary sclerosis cholangitis,^(^ ^36^ ^)^ the I148M variant was related to reduced survival. Patients with primary biliary cirrhosis carrying I148M were protected against cholestatic pruritus, whereas a higher incidence of hepatic steatosis was reported in patients with Wilson disease^(^ ^37^ ^)^ and with inflammatory bowel disease.^(^ ^38^ ^)^

Protein structural analysis suggested that the aminoacidic substitution C>G might result in reduced accessibility of the substrate in the PNPLA3 catalytic site, thus supporting a loss‐of‐function mutation.^(^ ^39^ ^)^ A study conducted on human hepatocytes showed that the presence of *PNPLA3* I148M correlated with decreased release of very low density lipoprotein (VLDL), as a possible mechanism of PNPLA3‐induced liver steatosis in humans.^(^ ^40^ ^)^ To this end, lipidomic analysis revealed that increased fat accumulation in livers with the *PNPLA3* I148M was not associated with the prevalence of lipotoxic lipids, such as saturated fatty acids and ceramides, that usually represent the hallmark of “metabolic NAFLD.”^(^ ^41^ ^)^ Interestingly, *PNPLA3* I148M carriers are protected against cardiovascular diseases,^(^ ^42^ ^)^ despite the higher liver‐fat content. This might be explained by the higher retention of polyunsaturated fatty acids (PUFAs) in liver and decreased PUFA content in VLDL composition. Notably, hepatocytes expressing this genetic variant displayed accumulation of PUFAs in DGs, similar to cell knockout for PNPLA3.^(^ ^42^ ^)^ These intriguing data propose that PNPLA3 remodels PUFA content in TGs and DGs, whereas its genetic variant creates a predisposition for hepatic PUFA retention and therefore contributes to protection from insulin resistance and cardiovascular diseases.^(^ ^43^ ^)^ Notably, human *PNPLA3* I148M knock‐in mice develop hepatic steatosis only on a high‐sucrose diet and develop hepatic inflammation and fibrosis on a NASH diet.^(^ ^44^ ^)^ Other studies have shown that the diminished clearance of PNPLA3 I148M could be due to its decreased ubiquitination, and, therefore, defects in hepatic TG mobilization from LDs may derive from accumulation of I148M that lacks hydrolytic properties.^(^ ^45^ ^)^

Evidence highlighted the possible interaction between PNPLA3 and CGI‐58 in hepatocytes.^(^ ^7^ ^)^ In detail, co‐immunoprecipitation and pulldown experiments in hepatoma cells (HuH7) and mouse livers demonstrated that PNPLA3 I148M sequestrates CGI‐58, thus impeding its interaction with ATGL and therefore preventing LD depletion. Importantly, CGI‐58 is required for targeting PNPLA3 on LDs, as *CGI‐58*‐knockout hepatic cells lack PNPLA3 on their LDs.^(^ ^7^ ^)^

**FIG. 2:**
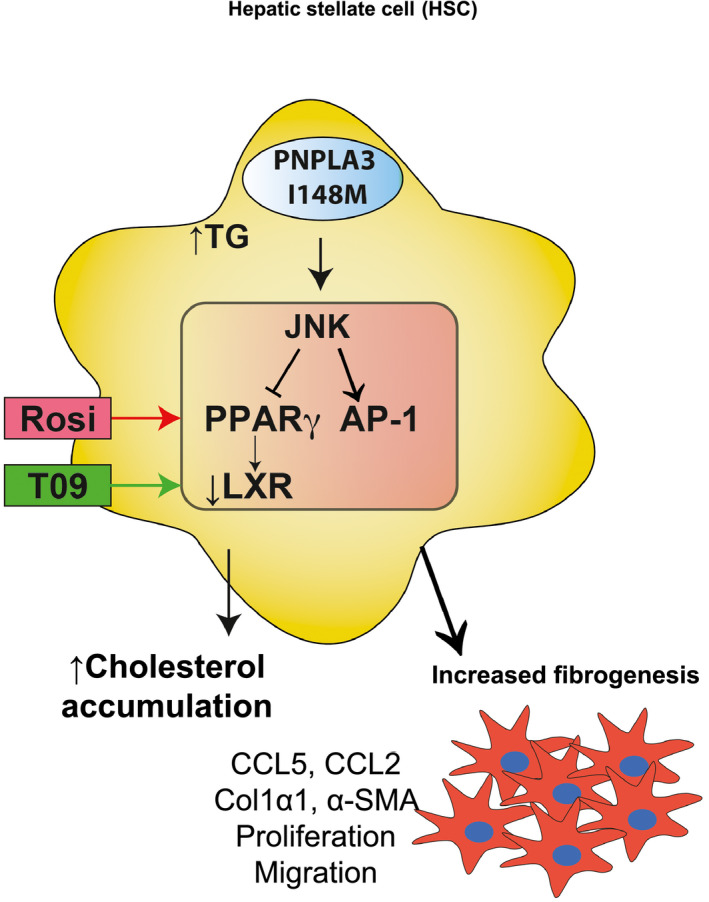
PNPLA3 I148M expressing HSCs show increased fibrogenic features. The PNPLA3 I148M mutation determines increased lipid accumulation, aberrant FFA composition, and reduced retinol content in HSCs. This upregulates JNK signaling, which phosphorylates PPARγ and thereby decreases its transcriptional activity. Lower PPARγ signaling promotes, on one hand, AP‐1–driven production of pro‐inflammatory cytokines, accompanied by immune‐cell recruitment; on the other hand, LXR down‐regulation results in free cholesterol accumulation and worsens profibrogenic features in HSCs. Restoring LXR activity by its pharmacological agonist T0901317 (green arrow), ameliorates cholesterol homeostasis and reduces aberrant activation of HSCs with the PNPLA3 variant, whereas the PPARγ agonist Rosi (red arrow) shows only partial antifibrogenic outcomes. Abbreviations: AP‐1, activator protein 1; CCL, chemokine (C‐C motif) ligand; Col1α1, collagen type 1, α1; FFA, free fatty acid; HSC, hepatic stellate cell; JNK, c‐Jun N‐terminal kinase; LXR, liver X receptor; PNPLA3, patatin‐like phospholipase domain–containing protein 3; PPARγ, peroxisome proliferator–activated receptor γ; Rosi, rosiglitazone; α‐SMA, α‐smooth muscle actin.

The *PNPLA3* variant has been associated with a higher risk of developing severe liver fibrosis, irrespective of its cause (i.e., NAFLD, ALD, or HCV).^(^ ^46^ ^)^ This association has stimulated mechanistic studies on the role of PNPLA3 in hepatic stellate cells (HSCs).^(^ ^47^ ^)^ Notably, PNPLA3 is induced in human tissue staining from NASH patients,^(^ ^48^ ^)^ and it is necessary for acquisition/maintenance of the myofibroblast‐like phenotype in human HSCs.^(^ ^47^ ^)^ Furthermore, its genetic variant, I148M, results in exacerbated inflammatory and fibrogenic features of HSCs (i.e., migration and proliferation) as a result of decreased PPARγ signaling and subsequent hyperactivation of the c‐Jun N‐terminal kinase/activator protein 1 pathway^(^ ^47^, ^49^ ^)^ (Fig. 2). Furthermore, decreased PPARγ results in down‐regulation of LXR, thus leading to impairment of cholesterol homeostasis in HSCs with the genetic variant of *PNPLA3*.^(^ ^50^ ^)^ Interestingly, only the pharmacological activation of LXR by its synthetic agonist T0901317, and not of PPARγ by rosiglitazone, reduced sustained HSC activation^(^ ^50^ ^)^ (Fig. 2). Targeting HSC and NR signaling may therefore represent a possible therapeutic intervention for limiting fibrosis development and progression in patients carrying the I148M variant, but this needs to be validated *in vivo* in future studies. Inhibition strategies have recently introduced a possible therapeutic approach for decreasing deleterious PNPLA3 I148M actions in livers. ASO‐mediated silencing of PNPLA3 reduced liver steatosis and inflammation in mice overexpressing the *PNPLA3* variant and fed a high‐sucrose diet.^(^ ^51^ ^)^ These observations might encourage personalized therapeutic strategies based on reduction of the I148M allele with specific ASOs,^(^ ^44^ ^)^ which is also supported by latest data obtained on target gene therapy on proprotein convertase subtilisin/kexin type 9.^(^ ^52^ ^)^ Along with the RNA interference approach, a parallel translational prospective approach might be represented by the increase of PNPLA3 I148M degradation, to avoid its critical accumulation on LDs of hepatocytes. By using a strategy called proteolysis‐targeting chimera of Halo‐tagged PNPLA3 to accelerate its proteasomal degradation, Smagris and colleagues^(^ ^44^ ^)^ obtained a successful normalization of hepatic TG content in mice overexpressing the *PNPLA3* genetic variant. Another way to interfere with PNPLA3 I148M might be pursued by targeting the close partnership between PNPLA3 and CGI‐58, as it has been shown that this interaction can be modulated by FFAs and CGI‐58 synthetic ligands.^(^ ^53^ ^)^

Although its enzymatic activity remains enigmatic, *PNPLA3* I148M represents an important emerging prognostic marker for risk stratification and a potential therapeutic target because of its major implications for the risk of progression toward more advanced fibrosis and HCC in virtually all major hepatic disorders.

## HSL, a Multifunctional Pleiotropic Lipase

HSL is an enzyme of relatively broad specificity, which has the ability to hydrolyze TGs, DGs, and MGs, as well as cholesterol and retinyl esters, with no phospholipase activity.^(^ ^54^ ^)^ Phosphorylation of cyclic adenosine monophosphate–dependent protein kinase renders HSL hormone‐insensitive (although phosphorylation alone is not enough to activate it), leading to its redistribution from the cytoplasm to the LDs.^(^ ^55^ ^)^ Regulation of HSL in the adipocytes is the primary means by which lipolytic agents (such as catecholamines or insulin) stimulate the release of FFAs and control systemic plasma levels. Another way to control HSL is through the fatty acid binding protein 4.^(^ ^56^ ^)^ FFAs mobilized from intracellular stores through the action of HSL can be used in nonshivering thermogenesis as physiological activators of uncoupling protein 1 in BAT. Accordingly, HSL has been shown to retain high enzymatic activity at low temperatures.^(^ ^57^ ^)^ In addition to adipocytes, HSL is found in skeletal muscle, heart, brain, adrenal glands, testes, and macrophages. Its function toward cholesterol ester may be of importance in macrophages, where HSL was suggested to regulate foam cell formation.^(^ ^58^ ^)^ Furthermore, the presence of HSL in adrenal and reproductive tissues suggests a role in steroidogenesis and in sperm development, by regulating the availability of free cholesterol.^(^ ^59^ ^)^ In this regard, Osuga et al.^(^ ^60^ ^)^ showed that mice lacking HSL displayed oligospermia. Systemic HSL deficiency in mice resulted in fatty liver in an age‐dependent manner, whereas in the liver lack of HSL did not affect hepatic fat content.^(^ ^60^ ^)^ Conversely, mice with HSL knockout in WAT displayed fatty liver with inflammatory infiltrates, abnormal adipokine secretion, and systemic insulin resistance.^(^ ^61^ ^)^ Interestingly, adenoviral overexpression of HSL reduced hepatic TG levels in ob/ob and HFD‐fed mice, suggesting that hepatic HSL/ATGL might promote FFA oxidation, stimulate the release of FFAs, and ameliorate hepatic steatosis.^(^ ^62^ ^)^ Collectively, HSL covers a pleiotropic spectrum of activities toward different lipid molecules, showing multiorgan involvement in lipid metabolism and substrate generation.

Human studies have confirmed the existence of several SNPs: HSL promoter variant −60C>G was shown to significantly lower fasting nonesterified fatty acid levels and low‐density lipoprotein (LDL) cholesterol in carriers of this mutation versus noncarriers.^(^ ^63^ ^)^ Another study on the same genetic variation, −60C>G, evidenced male infertility.^(^ ^64^ ^)^ A frame‐shift deletion in the *Lipase E* gene, which encodes HSL, was found to increase insulin resistance and the risk of developing type 2 diabetes.^(^ ^65^ ^)^ Interestingly, the disruption of HSL in humans down‐regulated PPARγ‐responsive signaling pathways because of decreased production of endogenous ligands for NRs.^(^ ^65^ ^)^

## MGL as the Last Enzymatic Step in MG Degradation at the Crossroads Between Lipid and Cannabinoid Metabolism

MGL is the rate‐limiting enzyme of the FFA degradation pathway and hydrolyzes MGs deriving from phospholipids or TGs into glycerol and FFAs.^(^ ^66^ ^)^ Moreover, MGL hydrolyzes 2‐arachidonoylglycerol, a potent ligand within the endocannabinoid system, into arachidonic acid (AA). MGL is expressed both in brain and peripheral tissues, such as kidney, ovary, testis, adrenal gland, adipose tissue, and heart tissues.^(^ ^3^ ^)^ At the cellular level, MGL is located in the cytoplasm, plasma membrane, and LDs.^(^ ^3^ ^)^ Metabolic studies have highlighted that when *Mgl* ^−/−^ animals were challenged with an HFD, liver levels of MG species were highly increased, along with diminished weight gain,^(^ ^67^ ^)^ better insulin sensitivity, and better glucose tolerance.^(^ ^68^ ^)^ Although previous reports have demonstrated a key role of MGL in tumor growth/oncogenic signaling, such as invasion and migration,^(^ ^69^ ^)^ others have shown that MGL deletion resulted in colorectal‐cancer growth inhibition.^(^ ^70^ ^)^ Xiang et al.^(^ ^71^ ^)^ demonstrated that in tumor‐associated macrophages, MGL deficiency resulted in lipid overload. Mechanistically, MGL deficiency was shown to promote cannabinoid receptor 2/toll‐like receptor 4–dependent macrophage activation, which further suppresses the recruitment of tumor‐associated CD8^+^ T cells.^(^ ^71^ ^)^

**FIG. 3:**
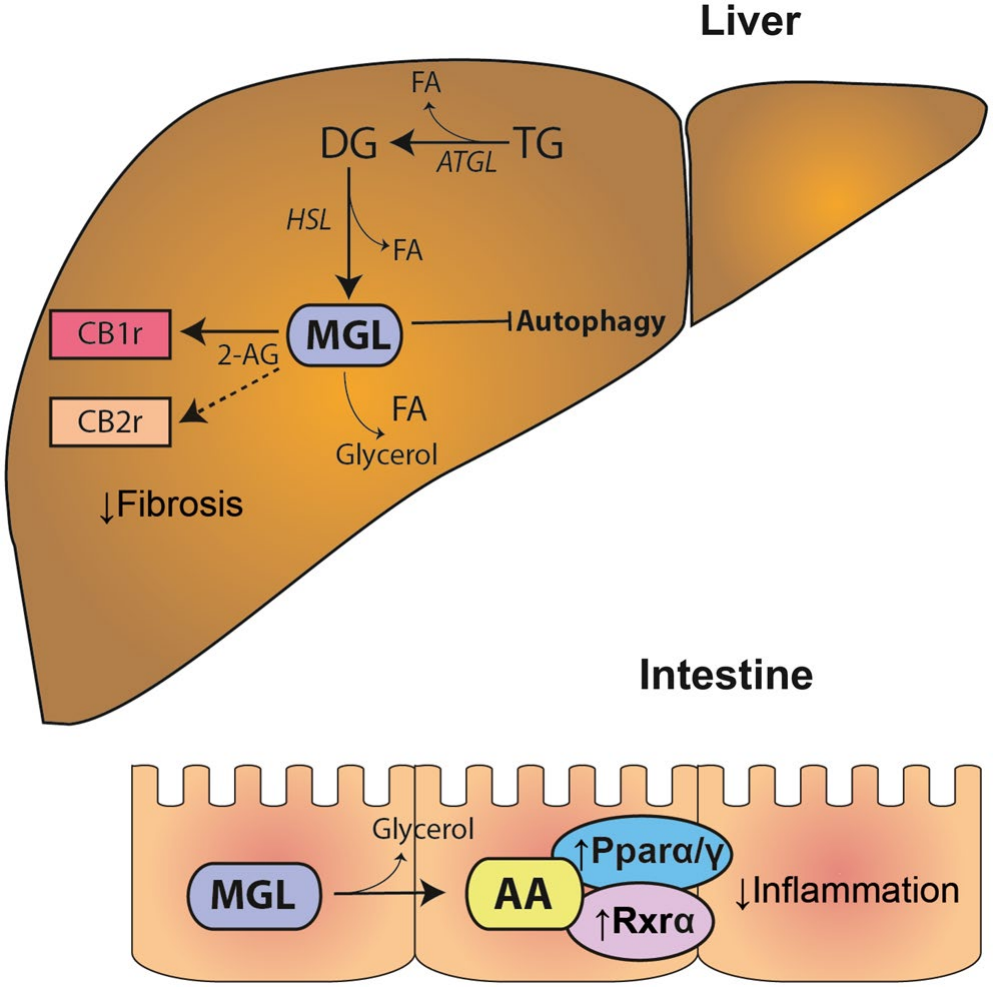
MGL at the crossroads between the cannabinoid and lipid signaling pathways. MGL is the major enzyme degrading the endogenous cannabinoid ligand 2‐AG, which in turn can bind either CB1r or CB2r, connected with fibrosis development. In the liver, MGL inhibition was shown to decrease autophagy of immune cells, resulting in decreased fibrosis development. MGL hydrolyzes 2‐AG into AA, a precursor of prostaglandin synthesis and a ligand of several NRs (PPARα, PPARγ, and RXR). In the intestine, lack of MGL was shown to be protective, as it reduces local inflammation. Abbreviations: 2‐AG, 2‐arachidonoylglycerol; AA, arachidonic acid; CB1r, cannabinoid receptor 1; CB2r, cannabinoid receptor 2; FA, fatty acid; HSL, hormone‐sensitive lipase; MGL, monoacylglycerol lipase; NR, nuclear receptor; PPAR, peroxisome proliferator–activated receptor; RXR, retinoid X receptor.

In the liver, MGL inhibition limits LPS‐induced inflammation; its global genetic and pharmacological invalidation protects against liver lesions induced by ischemia/reperfusion injury.^(^ ^72^ ^)^ In a microarray study, the expression of MGL was found to be higher in HCC tumors than in matched tissues,^(^ ^73^ ^)^ promoting cell growth and invasion. Recently, the effect of MGL deficiency on fibrosis development was investigated.^(^ ^74^ ^)^ Lack of MGL promoted fibrosis regression due to autophagy‐mediated anti‐inflammatory properties in macrophages.^(^ ^74^ ^)^ In cholestatic liver disease, MGL invalidation was shown to be protective, as it was able to rescue the mitochondrial respiration/antioxidant response and ameliorate cholestasis.^(^ ^75^ ^)^ Moreover, AA accumulation in the intestine reduced inflammation through retinoid X receptor binding and activation of PPARs and FXR competition.^(^ ^75^ ^)^ Strikingly, the role of MGL in several liver diseases remains unappreciated, and further investigations are warranted (Fig. 3).

Human SNP studies showed that four *MGL* SNPs (rs782440, rs13076593, rs549662, and rs541855) were associated with increased LDL particle size and that rs3773159 was associated with type 2 diabetes,^(^ ^76^ ^)^ whereas *MGL* intron 3 locus‐variant (rs684358) was associated with high body mass index.^(^ ^77^ ^)^

## Carboxylesterase Gene Family and Their Role in Lipolysis of Luminal LDs

More recently, members from the mammalian carboxylesterase (*CES*) gene family were demonstrated to play an important role in hepatic lipid metabolism and energy homeostasis.^(^ ^78^ ^)^ Although, ATGL and HSL are the major lipases in TG (and cholesteryl ester) hydrolysis of cytosolic LDs, *CES* family members may play a role in lipolysis of luminal LDs of the ER.^(^ ^79^ ^)^ TG hydrolysis of luminal LDs has been suggested to deliver substrates for VLDL‐TG production, in addition to lipid transfer through the microsomal TG transfer protein.^(^ ^79^ ^)^ In humans, two predominant isoforms (CES1 and CES2) have been studied for their metabolism of drugs and toxicants.^(^ ^80^ ^)^ Reduced hepatic expression of CES2 has been observed in patients with NASH.^(^ ^81^ ^)^ In addition, activity‐based protein profiling revealed reduced activity of CES2 in liver biopsies of humans with obesity.^(^ ^81^ ^)^ In mice, the gene *Ces2c* was found to be located in the ER lumen and highly expressed in the liver and intestine^(^ ^78^ ^)^; reduced hepatic *Ces2c* expression was linked to steatosis and insulin resistance.^(^ ^82^ ^)^ Cell preparations enriched with Ces2 protein members demonstrated substantial TG hydrolytic activities for *Ces2a* and *Ces2c*.^(^ ^78^ ^)^ *Ces2c* overexpression leads to increased fatty acid oxidation and protection from HFD‐induced NAFLD.^(^ ^83^ ^)^ In contrast, knockdown of hepatic *Ces2c* provoked hepatic steatosis through ER stress–linked induction of lipogenesis.^(^ ^78^ ^)^ Interestingly, *Ces2c* expression is highest in the duodenum, indicating that it may play a role in intestinal lipid catabolism and lipoprotein production.^(^ ^84^ ^)^

## Conclusions

Normal lipolytic activity is crucial for energy homeostasis in different tissues, providing pivotal FFA mediators, which are involved in a complex spectrum of metabolic pathways. Although important steps were taken toward understanding the complex regulation underlying the lipid network and signaling, many questions still remain unanswered. In addition, hepatic lipases and endothelial lipases represent further extracellular enzymes that both hydrolyze TG and phospholipids and display potentially overlapping/complementary roles in lipoprotein metabolism. A better characterization of the lipid‐catalyzing machinery might lead to therapeutic strategies aimed at targeting/reprogramming lipid metabolism and treating metabolic disorders.

## Author Contributions

M.T. and M. Trauner drafted the concept of the review article. M.T. and F.V.B. wrote the article. M. Trauner critically revised and corrected the article for final approval.

## References

[1] Lass A , Zimmermann R , Oberer M , Zechner R . Lipolysis ‐ a highly regulated multi‐enzyme complex mediates the catabolism of cellular fat stores. Prog Lipid Res 2011;50:14‐27. 21087632 10.1016/j.plipres.2010.10.004 PMC3031774

[2] Giammanco A , Cefalù AB , Noto D , Averna MR . The pathophysiology of intestinal lipoprotein production. Front Physiol 2015;6:61 10.3389/fphys.2015.00061. https://www.frontiersin.org/journals/physiology. 25852563 PMC4367171

[3] Zechner R , Zimmermann R , Eichmann TO , Kohlwein SD , Haemmerle G , Lass A , et al. FAT SIGNALS‐‐lipases and lipolysis in lipid metabolism and signaling. Cell Metab 2012;15:279‐291. 22405066 10.1016/j.cmet.2011.12.018 PMC3314979

[4] Dubland JA , Francis GA . Lysosomal acid lipase: at the crossroads of normal and atherogenic cholesterol metabolism. Front Cell Dev Biol 2015;3:3 10.3389/fcell.2015.00003. https://www.frontiersin.org/journals/cell‐and‐developmental‐biology. 25699256 PMC4313778

[5] Papackova Z , Cahova M . Fatty acid signaling: the new function of intracellular lipases. Int J Mol Sci 2015;16:3831‐3855. 25674855 10.3390/ijms16023831 PMC4346929

[6] Kienesberger PC , Oberer M , Lass A , Zechner R . Mammalian patatin domain containing proteins: a family with diverse lipolytic activities involved in multiple biological functions. J Lipid Res 2009;50(Suppl.):S63‐S68. 19029121 10.1194/jlr.R800082-JLR200 PMC2674697

[7] Wang Y , Kory N , BasuRay S , Cohen JC , Hobbs HH . PNPLA3, CGI‐58, and inhibition of hepatic triglyceride hydrolysis in mice. Hepatology 2019;69:2427‐2441. 30802989 10.1002/hep.30583 PMC6563103

[8] Osuga JI , Ishibashi S , Oka T , Yagyu H , Tozawa R , Fujimoto A , et al. Targeted disruption of hormone‐sensitive lipase results in male sterility and adipocyte hypertrophy, but not in obesity. Proc Natl Acad Sci U S A 2000;97:787‐792. 10639158 10.1073/pnas.97.2.787 PMC15409

[9] Lass A , Zimmermann R , Haemmerle G , Riederer M , Schoiswohl G , Schweiger M , et al. Adipose triglyceride lipase‐mediated lipolysis of cellular fat stores is activated by CGI‐58 and defective in Chanarin‐Dorfman syndrome. Cell Metab 2006;3:309‐319. 16679289 10.1016/j.cmet.2006.03.005

[10] Lord CC , Ferguson D , Thomas G , Brown AL , Rebecca C , Burrows A , et al. Metabolism does not require Atgl co‐activation. Cell Rep 2016;16:939‐949. 27396333 10.1016/j.celrep.2016.06.049 PMC5267558

[11] Listenberger LL , Ostermeyer‐Fay AG , Goldberg EB , Brown WJ , Brown DA . Adipocyte differentiation‐related protein reduces the lipid droplet association of adipose triglyceride lipase and slows triacylglycerol turnover. J Lipid Res 2007;48:2751‐2761. 17872589 10.1194/jlr.M700359-JLR200

[12] Lu X , Yang X , Liu J . Differential control of ATGL‐mediated lipid droplet degradation by CGI‐58 and G0S2. Cell Cycle 2010;9:2719‐2725. 20676045 10.4161/cc.9.14.12181 PMC3040957

[13] Haemmerle G , Lass A , Zimmermann R , Gorkiewicz G , Meyer C , Rozman J , et al. Defective lipolysis and altered energy metabolism in mice lacking adipose triglyceride lipase. Science 2006;312:734‐737. 16675698 10.1126/science.1123965

[14] Fuchs CD , Claudel T , Kumari P , Haemmerle G , Pollheimer MJ , Stojakovic T , et al. Absence of adipose triglyceride lipase protects from hepatic endoplasmic reticulum stress in mice. Hepatology 2012;56:270‐280. 22271167 10.1002/hep.25601

[15] Ong KT , Mashek MT , Bu SY , Mashek DG . Hepatic ATGL knockdown uncouples glucose intolerance from liver TAG accumulation. FASEB J 2013;27:313‐321. 22993196 10.1096/fj.12-213454 PMC3528322

[16] Ong KT , Mashek MT , Bu SY , Greenberg AS , Mashek DG . Adipose triglyceride lipase is a major hepatic lipase that regulates triacylglycerol turnover and fatty acid signaling and partitioning. Hepatology 2011;53:116‐126. 20967758 10.1002/hep.24006 PMC3025059

[17] Jha P , Claudel T , Baghdasaryan A , Mueller M , Halilbasic E , Das SK , et al. Role of adipose triglyceride lipase (PNPLA2) in protection from hepatic inflammation in mouse models of steatohepatitis and endotoxemia. Hepatology 2014;59:858‐869. 24002947 10.1002/hep.26732

[18] Schweiger M , Romauch M , Schreiber R , Grabner GF , Hütter S , Kotzbeck P , et al. Pharmacological inhibition of adipose triglyceride lipase corrects high‐fat diet‐induced insulin resistance and hepatosteatosis in mice. Nat Commun 2017;8:14859 10.1038/ncomms14859. https://www.nature.com/ncomms/. 28327588 PMC5364409

[19] Brown JM , Betters JL , Lord C , Ma Y , Han X , Yang K , et al. CGI‐58 knockdown in mice causes hepatic steatosis but prevents diet‐induced obesity and glucose intolerance. J Lipid Res 2010;51:3306‐3315. 20802159 10.1194/jlr.M010256 PMC2952571

[20] Coassin S , Schweiger M , Kloss‐Brandstätter A , Lamina C , Haun M , Erhart G , et al. Investigation and functional characterization of rare genetic variants in the adipose triglyceride lipase in a large healthy working population. PLoS Genet 2010;6:e1001239 10.1371/journal.pgen.1001239. https://journals.plos.org/plosgenetics/. 21170305 PMC3000363

[21] Pennisi EM , Missaglia S , Dimauro S , Bernardi C , Akman HO , Tavian D . A myopathy with unusual features caused by PNPLA2 gene mutations. Muscle Nerve 2015;51:609‐613. 25287355 10.1002/mus.24477

[22] Schweiger M , Lass A , Zimmermann R , Eichmann TO , Zechner R . Neutral lipid storage disease: genetic disorders caused by mutations in adipose triglyceride lipase/PNPLA2 or CGI‐58/ABHD5. Am J Physiol Endocrinol Metab 2009;297:E289‐E296. 19401457 10.1152/ajpendo.00099.2009

[23] Chamoun Z , Vacca F , Parton RG , Gruenberg J . PNPLA3/adiponutrin functions in lipid droplet formation. Biol Cell 2013;105:219‐233. 23398201 10.1111/boc.201200036

[24] Baulande S , Lasnier F , Lucas M , Pairault J . Adiponutrin, a transmembrane protein corresponding to a novel dietary‐ and obesity‐linked mRNA specifically expressed in the adipose lineage. J Biol Chem 2001;276:33336‐33344. 11431482 10.1074/jbc.M105193200

[25] Kumari M , Schoiswohl G , Chitraju C , Paar M , Cornaciu I , Rangrez AY , et al. Adiponutrin functions as a nutritionally regulated lysophosphatidic acid acyltransferase. Cell Metab 2012;15:691‐702. 22560221 10.1016/j.cmet.2012.04.008 PMC3361708

[26] Dubuquoy C , Robichon C , Lasnier F , Langlois C , Dugail I , Foufelle F , et al. Distinct regulation of adiponutrin/PNPLA3 gene expression by the transcription factors ChREBP and SREBP1c in mouse and human hepatocytes. J Hepatol 2011;55:145‐153. 21145868 10.1016/j.jhep.2010.10.024

[27] Huang Y , He S , Li JZ , Seo Y‐K , Osborne TF , Cohen JC , et al. A feed‐forward loop amplifies nutritional regulation of PNPLA3. Proc Natl Acad Sci U S A 2010;107:7892‐7897. 20385813 10.1073/pnas.1003585107 PMC2867902

[28] Chen W , Chang B , Li L , Chan L . Patatin‐like phospholipase domain‐containing 3/adiponutrin deficiency in mice is not associated with fatty liver disease. Hepatology 2010;52:1134‐1142. 20648554 10.1002/hep.23812 PMC2932863

[29] Bruschi FV , Tardelli M , Claudel T , Trauner M . PNPLA3 expression and its impact on the liver: current perspectives. Hepatic Med 2017;9:55‐66. 10.2147/HMER.S125718 PMC5683790 29158695

[30] Stickel F , Buch S , Lau K , Zu Schwabedissen HM , Berg T , Ridinger M , et al. Genetic variation in the PNPLA3 gene is associated with alcoholic liver injury in Caucasians. Hepatology 2011;53:86‐95. 21254164 10.1002/hep.24017

[31] Liu YL , Patman GL , Leathart JB , Piguet AC , Burt AD , Dufour JF , et al. Carriage of the PNPLA3 rs738409 C>G polymorphism confers an increased risk of non‐alcoholic fatty liver disease associated hepatocellular carcinoma. J Hepatol 2014;61:75‐81. 24607626 10.1016/j.jhep.2014.02.030

[32] Grimaudo S , Pipitone RM , Pennisi G , Celsa C , Cammà C , Di Marco V , et al. Association between PNPLA3 rs738409 C>G variant and liver‐related outcomes in patients with nonalcoholic fatty liver disease. Clin Gastroenterol Hepatol 2020;18:935‐944.e3. 31419571 10.1016/j.cgh.2019.08.011

[33] De Nicola S , Dongiovanni P , Aghemo A , Cheroni C , D’Ambrosio R , Pedrazzini M , et al. Interaction between PNPLA3 I148M variant and age at infection in determining fibrosis progression in chronic hepatitis C. PLoS One 2014;9:e106022 10.1371/journal.pone.010602. https://journals.plos.org/plosone/. 25171251 PMC4149487

[34] Chromy D , Mandorfer M , Bucsics T , Schwabl P , Bauer D , Scheiner B , et al. Prevalence and predictors of hepatic steatosis in patients with HIV/HCV coinfection and the impact of HCV eradication. AIDS Patient Care STDS 2019;33:197‐206. 31067123 10.1089/apc.2018.0333

[35] Viganò M , Valenti L , Lampertico P , Facchetti F , Motta BM , D’Ambrosio R , et al. Patatin‐like phospholipase domain‐containing 3 I148M affects liver steatosis in patients with chronic hepatitis B. Hepatology 2013;58:1245‐1252. 23564580 10.1002/hep.26445

[36] Friedrich K , Rupp C , Hov JR , Steinebrunner N , Weiss KH , Stiehl A , et al. A Frequent PNPLA3 Variant Is a Sex Specific Disease Modifier in PSC Patients with Bile Duct Stenosis. PLoS One 2013;8:e58734 10.1371/journal.pone.0058734. https://journals.plos.org/plosone/. 23505555 PMC3591368

[37] Stättermayer AF , Traussnigg S , Dienes HP , Aigner E , Stauber R , Lackner K , et al. Hepatic steatosis in Wilson disease ‐ role of copper and PNPLA3 mutations. J Hepatol 2015;63:156‐163. 25678388 10.1016/j.jhep.2015.01.034

[38] Mancina RM , Spagnuolo R , Milano M , Brogneri S , Morrone A , Cosco C , et al. PNPLA3 148M carriers with inflammatory bowel diseases have higher susceptibility to hepatic steatosis and higher liver enzymes. Inflamm Bowel Dis 2016;22:134‐140. 26355465 10.1097/MIB.0000000000000569 PMC4894778

[39] He S , McPhaul C , Li JZ , Garuti R , Kinch L , Grishin NV , et al. A sequence variation (I148M) in PNPLA3 associated with nonalcoholic fatty liver disease disrupts triglyceride hydrolysis. J Biol Chem 2010;285:6706‐6715. 20034933 10.1074/jbc.M109.064501 PMC2825465

[40] Pirazzi C , Adiels M , Burza MA , Mancina RM , Levin M , Ståhlman M , et al. Patatin‐like phospholipase domain‐containing 3 (PNPLA3) I148M (rs738409) affects hepatic VLDL secretion in humans and in vitro. J Hepatol 2012;57:1276‐1282. 22878467 10.1016/j.jhep.2012.07.030

[41] Luukkonen PK , Zhou Y , Sädevirta S , Leivonen M , Arola J , Orešič M , et al. Hepatic ceramides dissociate steatosis and insulin resistance in patients with non‐alcoholic fatty liver disease. J Hepatol 2016;64:1167‐1175. 26780287 10.1016/j.jhep.2016.01.002

[42] Simons N , Isaacs A , Koek GH , Kuč S , Schaper NC , Brouwers MCGJ . PNPLA3, TM6SF2, and MBOAT7 genotypes and coronary artery disease. Gastroenterology 2017;152:912‐913. 28157516 10.1053/j.gastro.2016.12.020

[43] Luukkonen PK , Nick A , Hölttä‐Vuori M , Thiele C , Isokuortti E , Lallukka‐Brück S , et al. Human PNPLA3‐I148M variant increases hepatic retention of polyunsaturated fatty acids. JCI Insight 2019;4:127902 10.1172/jci.insight.127902. https://insight.jci.org/. 31434800 PMC6777808

[44] Smagris E , Basuray S , Li J , Huang Y , Lai KM , Gromada J , et al. Pnpla3I148M knockin mice accumulate PNPLA3 on lipid droplets and develop hepatic steatosis. Hepatology 2015;61:108‐118. 24917523 10.1002/hep.27242 PMC4262735

[45] BasuRay S , Smagris E , Cohen JC , Hobbs HH . The PNPLA3 variant associated with fatty liver disease (I148M) accumulates on lipid droplets by evading ubiquitylation. Hepatology 2017;66:1111‐1124. 28520213 10.1002/hep.29273 PMC5605398

[46] Trépo E , Romeo S , Zucman‐Rossi J , Nahon P . PNPLA3 gene in liver diseases. J Hepatol 2016;65:399‐412. 27038645 10.1016/j.jhep.2016.03.011

[47] Bruschi FV , Claudel T , Tardelli M , Caligiuri A , Stulnig TM , Marra F , et al. The PNPLA3 I148M variant modulates the fibrogenic phenotype of human hepatic stellate cells. Hepatology 2017;65:1875‐1890. 28073161 10.1002/hep.29041

[48] Bruschi FV , Tardelli M , Herac M , Claudel T , Trauner M . Metabolic regulation of hepatic PNPLA3 expression and severity of liver fibrosis in patients with NASH. Liver Int 2020; 10.1111/liv.14402. https://onlinelibrary.wiley.com/journal/14783231. PMC7318357 32043752

[49] Tardelli M , Bruschi FV , Claudel T , Moreno‐Viedma V , Halilbasic E , Marra F , et al. AQP3 is regulated by PPARγ and JNK in hepatic stellate cells carrying PNPLA3 I148M. Sci Rep 2017;7:14661 10.1038/s41598-017-14557-9. https://www.nature.com/srep/. 29116096 PMC5676689

[50] Bruschi FV , Claudel T , Tardelli M , Starlinger P , Marra F , Trauner M . PNPLA3 I148M variant impairs liver X receptor signaling and cholesterol homeostasis in human hepatic stellate cells. Hepatol Commun 2019;3:1191‐1204. 31497741 10.1002/hep4.1395 PMC6719741

[51] Lindén D , Ahnmark A , Pingitore P , Ciociola E , Ahlstedt I , Andréasson AC , et al. Pnpla3 silencing with antisense oligonucleotides ameliorates nonalcoholic steatohepatitis and fibrosis in Pnpla3 I148M knock‐in mice. Mol Metab 2019;22:49‐61. 30772256 10.1016/j.molmet.2019.01.013 PMC6437635

[52] Mullard A . PCSK9‐lowering RNAi contender clears first phase III trial. Nat Rev Drug Discov 2019;18:737 10.1038/d41573-019-00153-1. https://www.nature.com/nrd/. 31570849

[53] Yang A , Mottillo EP , Mladenovic‐Lucas L , Zhou L , Granneman JG . Dynamic interactions of ABHD5 with PNPLA3 regulate triacylglycerol metabolism in brown adipocytes. Nat Metab 2019;1:560‐569. 31497752 10.1038/s42255-019-0066-3 PMC6730670

[54] Lampidonis AD , Rogdakis E , Voutsinas GE , Stravopodis DJ . The resurgence of hormone‐sensitive lipase (HSL) in mammalian lipolysis. Gene 2011;477:1‐11. 21241784 10.1016/j.gene.2011.01.007

[55] McKnight GS , Cummings DE , Amieux PS , Sikorski MA , Brandon EP , Planas JV , et al. Cyclic AMP, PKA, and the physiological regulation of adiposity. Recent Prog Horm Res 1998;53:139‐159. 9769707

[56] Smith AJ , Thompson BR , Sanders MA , Bernlohr DA . Interaction of the adipocyte fatty acid‐binding protein with the hormone‐sensitive lipase: Regulation by fatty acids and phosphorylation. J Biol Chem 2007;282:32424‐32432. 17785468 10.1074/jbc.M703730200

[57] Langin D , Laurell H , Holst LS , Belfrage P , Holm C . Gene organization and primary structure of human hormone‐sensitive lipase: possible significance of a sequence homology with a lipase of Moraxella TA144, an antarctic bacterium. Proc Natl Acad Sci U S A 1993;90:4897‐4901. 8506334 10.1073/pnas.90.11.4897 PMC46620

[58] Escary JL , Choy HA , Reue K , Schotz MC . Hormone‐sensitive lipase overexpression increases cholesteryl ester hydrolysis in macrophage foam cells. Arterioscler Thromb Vasc Biol 1998;18:991‐998. 9633942 10.1161/01.atv.18.6.991

[59] Yeaman SJ . Hormone‐sensitive lipase ‐ a multipurpose enzyme in lipid metabolism. Biochim Biophys Acta 1990;1052:128‐132. 2182129 10.1016/0167-4889(90)90067-n

[60] Osuga J , Ishibashi S , Oka T , Yagyu H , Tozawa R , Fujimoto A , et al. Targeted disruption of hormone‐sensitive lipase results in male sterility and adipocyte hypertrophy, but not in obesity. Proc Natl Acad Sci U S A 2000;97:787‐792. 10639158 10.1073/pnas.97.2.787 PMC15409

[61] Xia B , Cai GH , Yang H , Wang SP , Mitchell GA , Wu JW . Adipose tissue deficiency of hormone‐sensitive lipase causes fatty liver in mice. PLoS Genet 2017;13:e1007110 10.1371/journal.pgen.1007110. https://journals.plos.org/plosgenetics/. 29232702 PMC5741266

[62] Reid BN , Ables GP , Otlivanchik OA , Schoiswohl G , Zechner R , Blaner WS , et al. Hepatic overexpression of hormone‐sensitive lipase and adipose triglyceride lipase promotes fatty acid oxidation, stimulates direct release of free fatty acids, and ameliorates steatosis. J Biol Chem 2008;283:13087‐13099. 18337240 10.1074/jbc.M800533200 PMC2442319

[63] Talmud PJ , Palmen J , Luan J , Flavell D , Byrne CD , Waterworth DM , et al. Variation in the promoter of the human hormone sensitive lipase gene shows gender specific effects on insulin and lipid levels: Results from the Ely study. Biochim Biophys Acta 2001;1537:239‐244. 11731226 10.1016/s0925-4439(01)00076-x

[64] Vatannejad A , Khodadadi I , Amiri I , Vaisi‐Raygani A , Ghorbani M , Tavilani H . Genetic variation of hormone sensitive lipase and male infertility. Syst Biol Reprod Med 2011;57:288‐291. 21919688 10.3109/19396368.2011.608179

[65] Albert JS , Yerges‐Armstrong LM , Horenstein RB , Pollin TI , Sreenivasan UT , Chai S , et al. Null mutation in hormone‐sensitive lipase gene and risk of type 2 diabetes. N Engl J Med 2014;370:2307‐2315. 24848981 10.1056/NEJMoa1315496 PMC4096982

[66] Poursharifi P , Madiraju SRM , Prentki M . Monoacylglycerol signalling and ABHD6 in health and disease. Diabetes Obes Metab 2017;19(Suppl. 1):76‐89. 28880480 10.1111/dom.13008

[67] Tardelli M , Bruschi FV , Claudel T , Fuchs CD , Auer N , Kunczer V , et al. Lack of monoacylglycerol lipase prevents hepatic steatosis by favoring lipid storage in adipose tissue and intestinal malabsorption. J Lipid Res 2019;60:1284‐1292. 31048404 10.1194/jlr.M093369 PMC6602129

[68] Douglass JD , Zhou YX , Wu A , Zadrogra JA , Gajda AM , Lackey AI , et al. Global deletion of MGL in mice delays lipid absorption and alters energy homeostasis and diet‐induced obesity. J Lipid Res 2015;56:1153‐1171. 25842377 10.1194/jlr.M058586 PMC4442873

[69] Nomura DK , Long JZ , Niessen S , Hoover HS , Ng SW , Cravatt BF . Monoacylglycerol lipase regulates a fatty acid network that promotes cancer pathogenesis. Cell 2010;140:49‐61. 20079333 10.1016/j.cell.2009.11.027 PMC2885975

[70] Ye L , Zhang B , Seviour EG , Tao KX , Liu XH , Ling Y , et al. Monoacylglycerol lipase (MAGL) knockdown inhibits tumor cells growth in colorectal cancer. Cancer Lett 2011;307:6‐17. 21543155 10.1016/j.canlet.2011.03.007

[71] Xiang W , Shi R , Kang X , Zhang X , Chen P , Zhang L , et al. Monoacylglycerol lipase regulates cannabinoid receptor 2‐dependent macrophage activation and cancer progression. Nat Commun 2018;9:2574 10.1038/s41467-018-04999-8. https://www.nature.com/ncomms/. 29968710 PMC6030061

[72] Cao Z , Mulvihill MM , Mukhopadhyay P , Xu H , Erdélyi K , Hao E , et al. Monoacylglycerol lipase controls endocannabinoid and eicosanoid signaling and hepatic injury in mice. Gastroenterology 2013;144:808‐817. 23295443 10.1053/j.gastro.2012.12.028 PMC3608818

[73] Wang L , Zhu W , Zhao Y , Zhou J , Wang X , Pan Q , et al. Monoacylglycerol lipase promotes progression of hepatocellular carcinoma via NF‐κB‐mediated epithelial‐mesenchymal transition. J Hematol Oncol 2016;9:127 10.1186/s13045-016-0361-3. https://jhoonline.biomedcentral.com/. 27884159 PMC5123220

[74] Habib A , Chokr D , Wan J , Hegde P , Mabire M , Siebert M , et al. Inhibition of monoacylglycerol lipase, an anti‐inflammatory and antifibrogenic strategy in the liver. Gut 2018; 10.1136/gutjnl-2018-316137. https://gut.bmj.com/. 30301768

[75] Tardelli M , Bruschi FV , Fuchs CD , Claudel T , Auer N , Kunczer V , et al. Monoacylglycerol lipase inhibition protects from liver injury in mouse models of sclerosing cholangitis. Hepatology 2019; 10.1002/hep.30929. https://aasldpubs.onlinelibrary.wiley.com/journal/15273350. PMC7317927 31505038

[76] Rao P , Zhou Y , Ge SQ , Wang AX , Yu XW , Alzain MA , et al. Validation of type 2 diabetes risk variants identified by genome‐wide association studies in northern Han Chinese. Int J Environ Res Public Health 2016;13:E863 10.3390/ijerph13090863. https://www.mdpi.com/journal/ijerph. 27589775 PMC5036696

[77] Harismendy O , Bansal V , Bhatia G , Nakano M , Scott M , Wang X , et al. Population sequencing of two endocannabinoid metabolic genes identifies rare and common regulatory variants associated with extreme obesity and metabolite level. Genome Biol 2010;11:R118 10.1186/gb-2010-11-11-r118. https://genomebiology.biomedcentral.com/. 21118518 PMC3156957

[78] Lian J , Nelson R , Lehner R . Carboxylesterases in lipid metabolism: from mouse to human. Protein Cell 2018;9:178‐195. 28677105 10.1007/s13238-017-0437-z PMC5818367

[79] Hosokawa M . Structure and catalytic properties of carboxylesterase isozymes involved in metabolic activation of prodrugs. Molecules 2008;13:412‐431. 18305428 10.3390/molecules13020412 PMC6245361

[80] Fukami T , Kariya M , Kurokawa T , Iida A , Nakajima M . Comparison of substrate specificity among human arylacetamide deacetylase and carboxylesterases. Eur J Pharm Sci 2015;78:47‐53. 26164127 10.1016/j.ejps.2015.07.006

[81] Wang D , Zou L , Jin Q , Hou J , Ge G , Yang L . Human carboxylesterases: a comprehensive review. Acta Pharm Sin B 2018;8:699‐712. 30245959 10.1016/j.apsb.2018.05.005 PMC6146386

[82] Li Y , Zalzala M , Jadhav K , Xu Y , Kasumov T , Yin L , et al. Carboxylesterase 2 prevents liver steatosis by modulating lipolysis, endoplasmic reticulum stress, and lipogenesis and is regulated by hepatocyte nuclear factor 4 alpha in mice. Hepatology 2016;63:1860‐1874. 26806650 10.1002/hep.28472 PMC4874867

[83] Ruby MA , Massart J , Hunerdosse DM , Schönke M , Correia JC , Louie SM , et al. Human carboxylesterase 2 reverses obesity‐induced diacylglycerol accumulation and glucose intolerance. Cell Rep 2017;18:636‐646. 28099843 10.1016/j.celrep.2016.12.070 PMC5276805

[84] Chen YT , Trzoss L , Yang D , Yan B . Ontogenic expression of human carboxylesterase‐2 and cytochrome P450 3A4 in liver and duodenum: Postnatal surge and organ‐dependent regulation. Toxicology 2015;330:55‐61. 25724353 10.1016/j.tox.2015.02.007 PMC4496585

